# Genomic Insights Into Plant-Growth-Promoting Potentialities of the Genus *Frankia*

**DOI:** 10.3389/fmicb.2019.01457

**Published:** 2019-07-04

**Authors:** Imen Nouioui, Carlos Cortés-albayay, Lorena Carro, Jean Franco Castro, Maher Gtari, Faten Ghodhbane-Gtari, Hans-Peter Klenk, Louis S. Tisa, Vartul Sangal, Michael Goodfellow

**Affiliations:** ^1^School of Natural and Environmental Sciences, Faculty of Science, Agriculture and Engineering, Newcastle University, Newcastle upon Tyne, United Kingdom; ^2^Microbiology and Genetics Department, Universidad de Salamanca, Salamanca, Spain; ^3^The Chilean Collection of Microbial Genetic Resources (CChRGM), Instituto de Investigaciones Agropecuarias (INIA) – Quilamapu, Chillán, Chile; ^4^Institut National des Sciences Appliquées et de Technologie, Université de Carthage Centre Urbain Nord, Tunis, Tunisia; ^5^Laboratoire Microorganismes et Biomolécules Actives, Faculté de Sciences de Tunis, Université de Tunis El Manar, Tunis, Tunisia; ^6^Department of Molecular, Cellular and Biomedical Sciences, University of New Hampshire, Durham, NH, United States; ^7^Faculty of Health and Life Sciences, Northumbria University, Newcastle upon Tyne, United Kingdom

**Keywords:** PGPB, actinobacteria, biotic and abiotic stress, symbiosis, biofertilizers

## Abstract

This study was designed to determine the plant growth promoting (PGP) potential of members of the genus *Frankia*. To this end, the genomes of 21 representative strains were examined for genes associated directly or indirectly with plant growth. All of the *Frankia* genomes contained genes that encoded for products associated with the biosynthesis of auxins [indole-3-glycerol phosphate synthases, anthranilate phosphoribosyltransferases (*trp*D), anthranilate synthases, and aminases (*trp*A and B)], cytokinins (11 well-conserved genes within the predicted biosynthetic gene cluster), siderophores, and nitrogenases (*nif* operon except for atypical *Frankia*) as well as genes that modulate the effects of biotic and abiotic environmental stress (e.g., alkyl hydroperoxide reductases, aquaporin Z, heat shock proteins). In contrast, other genes were associated with strains assigned to one or more of four host-specific clusters. The genes encoding for phosphate solubilization (e.g., low-affinity inorganic phosphate transporters) and lytic enzymes (e.g., cellulases) were found in *Frankia* cluster 1 genomes, while other genes were found only in cluster 3 genomes (e.g., alkaline phosphatases, extracellular endoglucanases, pectate lyases) or cluster 4 and subcluster 1c genomes (e.g., NAD(P) transhydrogenase genes). Genes encoding for chitinases were found only in the genomes of the type strains of *Frankia casuarinae*, *F. inefficax*, *F. irregularis*, and *F. saprophytica*. In short, these *in silico* genome analyses provide an insight into the PGP abilities of *Frankia* strains of known taxonomic provenance. This is the first study designed to establish the underlying genetic basis of cytokinin production in *Frankia* strains. Also, the discovery of additional genes in the biosynthetic gene cluster involved in cytokinin production opens up the prospect that *Frankia* may have novel molecular mechanisms for cytokinin biosynthesis.

## Introduction

Actinobacteria classified in the genus *Frankia* (Brunchorst, 1886) are well known for their ability to induce nitrogen-fixing nodules in over 200 species of dicotyledonous (actinorhizal) plants representing eight angiosperm families (Normand et al., 2014). Mutualistic associations with *Frankia* strains allow actinorhizal plants to colonize extreme habitats, such as arid, nutritionally poor saline soils (Karthikeyan et al., 2009; Ngom et al., 2016b; Oshone et al., 2017). Until recently, the prospect of selecting *Frankia* strains for targeted growth promotion of actinorhizal plants in marginal soils was bedeviled by the difficulty of growing these slow-growing bacteria and by the poor state of their systematics. These obstacles have been addressed by the application of better methods for cultivating *Frankia* strains and by marked improvements in their systematics, mainly due to the application of genome sequence-based taxonomic procedures (Nouioui et al., 2016). The genus currently encompasses 11 validly named species: *Frankia alni* (Nouioui et al., 2016), the type species, *Frankia asymbiotica* (Nouioui et al., 2017c), *Frankia canadensis* (Normand et al., 2018), *Frankia casuarinae* (Nouioui et al., 2016), *Frankia coriariae* (Gtari et al., 2015; Nouioui et al., 2017a), *Frankia discariae* (Nouioui et al., 2017d), *Frankia elaeagni* (Gtari et al., 2004; Nouioui et al., 2016), *Frankia inefficax* (Nouioui et al., 2017a), *Frankia irregularis* (Nouioui et al., 2018b), *Frankia saprophytica* (Nouioui et al., 2018a), and *Frankia torreyi* (Nouioui et al., 2019), with the prospect of more to come in the near future (Tisa et al., 2016).

*Frankia* type strains can be assigned to four clusters with distinct host ranges (Normand et al., 2007; Tisa et al., 2013). Cluster 1 strains nodulate host plants classified in the families *Betulaceae*, *Casuarinaceae* (apart from *Gymnostoma*), and *Myricaceae* and can be further divided into three subgroups; those assigned to subcluster 1a infect *Alnus*–*Myrica* species, subcluster 1b strains, such as strain ARgP5 (Normand et al., 2018), infect *Alnus* and *Myricaceae* species while subcluster 1c includes *Frankia* strains that infect *Allocasuarina* and *Casuarina* species (Normand et al., 1996). In turn, cluster 2 strains are associated with plants classified in the families *Coriariaceae*, *Datiscaceae*, and *Rosaceae* and the type genus *Ceanothus* of the family *Rhamnaceae* while those in cluster 3 infect host plants belonging to the families *Elaeagnaceae*, *Myricaceae*, and *Rhamnaceae* (except *Ceanothus*); the genus *Gymnostoma* and occasionally *Alnus* species. The fourth cluster encompasses strains isolated from actinorhizal nodules that are unable to either infect or re-establish effective nodulation in the plant from which they were isolated.

*Frankia* genome sequences generated from representatives of the four clusters are providing valuable insights into the biological properties of members of the genus *Frankia* (Tisa et al., 2013, 2016), including their potential as a source of novel bioactive compounds (Udwary et al., 2011; Ogasawara et al., 2015) and as biocontrol agents (Gopinathan, 1995). It is particularly interesting that the sizes of *Frankia* genomes correlate with both host specificity and biogeographic distribution (Normand et al., 2007; Tisa et al., 2013). Further improvements in *Frankia* systematics and the use of genomic data open up the prospect of selecting specific mutualistic associations between *Frankia* strains and their hosts for bioremediation (Richards et al., 2002; Diagne et al., 2013, 2015; Rehan et al., 2014a, b, 2015; Baker et al., 2015; Furnholm et al., 2017), notably for saline soils (Sasakawa, 2003; Ngom et al., 2016a; Oshone et al., 2017) and in enhancing the fertility of marginal land (Schwencke and Carú, 2001; Benson and Dawson, 2007; Ngom et al., 2016b).

Plant-growth-promoting bacteria (PGPB) are of interest in sustainable agricultural research and their beneficial effects on plants have been commercially exploited (Gonzalez et al., 2015). In contrast, relatively little is known about the plant growth promoting (PGP) properties of *Frankia* strains though some have been found to solubilize inorganic phosphate (Sayed et al., 2002) and to synthesize plant hormones (Hirsch et al., 1997; Péret et al., 2007) and siderophores (Boyer et al., 1999; Haansuu et al., 1999; Tisa et al., 2016). However, the improvements in *Frankia* systematics and the availability of full-genome sequences provide an opportunity to establish the distribution of PGP genes within the genomes of members of the genus and thereby their prospective roles in bioremediation. In the present study, the distribution of PGP genes within the genomes of representative *Frankia* strains was undertaken with particular reference to those associated with the synthesis of plant hormones, siderophores, and the regulation of phosphate metabolism.

## Materials and Methods

### Genome Sequences

Table 1 lists the source, host plant specificity, and genome accession numbers of 21 representative *Frankia* strains, including the type strains of *F. alni*, *F. asymbiotica*, *F casuarinae*, *F. coriariae*, *F discariae*, *F. elaeagni*, *F. inefficax*, *F. irregularis*, *F. saprophytica*, and *F. torryei*. The following seven type strains were included as outgroups: *Acidothermus cellulolyticus* 11B^T^, *Blastococcus saxobsidens* DD2^T^, *Geodermatophilus obscurus* G-20^T^, *Kineococcus radiotolerans* ATCC BAA-149^T^, *Modestobacter marinus* BC501, *Nakamurella multipartita* DSM 44233^T^, and *Sporichthya polymorpha* DSM 43042^T^. All of the genome sequences of these strains were obtained from GenBank (accession numbers: CP000481, FO117623, CP001867, CP000750, FO203431, CP001737, and AQZX00000000, respectively).

**TABLE 1:** Origin of *Frankia* strains and their genomic features.

**Strains**	**Origin of isolation**	**Genome accession number**	**Genome size**	**Total gene number**	Percentage of genes^*^	**References**
**Cluster 1**						
**Sub-cluster 1a**						
*Frankia alni* ACN14a^T^	*Alnus crispa*	CT573213	7.497934	6338	4.4	Nouioui et al.,2016
*Frankia torreyi* CpI1^T^	*Comptonia peregrina*	JYFN00000000	7.61955	6449	4.3	Nouioui et al.,2019
*Frankia*. *torreyi* ACN1^AG^	*A. crispa*	LJPA00000000	7.52105	6287	4.4	Baker et al., 1979; Lalonde et al.,1981
*Frankia*. sp. QA3	*Alnus nitida*	CM001489	7.59085	6366	4.4	Hafeez et al.,1984
**Sub-cluster 1c**						
*Frankia casuarinae* CcI3	*Casuarina cunninghamiana*	CP000249	5.433628	5060	5.5	Nouioui et al.,2016
*F. casuarinae* Allo2	*Allocasuarina*	JPHT00000000	5.35211	4738	5.8	Girgis and Schwencke,1993
*F. casuarinae* BMG5.23	*Casuarina glauca*	NZ_JDWE00000000	5.26596	4608	6.0	Ghodhbane-Gtari et al.,2010
*F. casuarinae* CcI6	*C. cunninghamiana*	AYTZ00000000	5.57578	4780	5.8	Mansour and Moussa,2005
*F. casuarinae* CeD	*Casuarina equisetifolia*	JPGU00000000	5.0046	4350	6.4	Diem and Dommergues,1983
*F. casuarinae* Thr	*C. cunninghamiana*	JENI00000000	5.309833	4931	5.6	Girgis et al.,1990
**Cluster 2**						
*Frankia coriariae* BMG5.1^T^	*Coriaria myrtifolia*	JWIO00000000	5.795263	5403	5.1	Gtari et al., 2015; Nouioui et al.,2017b
Candidatus Frankia datiscae Dg1	*Datisca glomerata*	CP002801	5.323186	4799	5.8	Persson et al.,2011
**Cluster 3**						
*Frankia elaeagni* BMG5.12^T^	*Elaeagnus angustifolia*	ARFH00000000	7.589313	6386	4.3	Gtari et al., 2004; Nouioui et al.,2013, 2016
*Frankia discariae* BCU110501^T^	*Discaria trinervis*	ARDT00000000	7.891711	6845	4.0	Nouioui et al.,2017d
*Frankia* sp. EUN1f	*Elaeagnus umbellata*	ADGX00000000	9.35274	7942	3.5	Lalonde et al.,1981
*Frankia* sp. EAN1pec	*E. angustifolia*	CP000820	8.98204	7542	3.6	
*Frankia irregularis* DSM 45899^T^	*C. equisetifolia*	FAOZ00000000	9.537992	8018	3.4	Nouioui et al.,2018b
*Frankia* sp. R43	*C. cunninghamiana*	LFCW00000000	10.4489	8464	3.3	Zhang et al., 1984; Lechevalier,1986
**Cluster 4**						
*Frankia saprophytica* CN3^T^	*Coriaria nepalensis*	AGJN00000000	9.978592	8452	3.3	Nouioui et al.,2018a
*Frankia inefficax* EuI1c^T^	*E. umbellata*	CP002299	8.815781	7376	3.7	Nouioui et al.,2017a
*Frankia* sp. DC12	*Datisca cannabina*	LANG00000000	6.88434	5743	4.8	Hafeez, 1983; Hameed et al.,1994

^*^Percentage of genes used for dendrogram construction.

### *In silico* Screening of PGP Genes

The genomes of the 21 *Frankia* strains were annotated using the Rapid Annotation Subsystem Technology server (RAST) (Aziz et al., 2008, 2012). The distribution of PGP genes in the genomes was determined using the SEED server (Overbeek et al., 2014) with a focus on genes encoding for nitrogen fixation, phosphate solubilization, plant hormones, siderophores, lytic enzymes, and those modulating the effect of environmental stress. The gene clusters of the nitrogenase complex (*nif*) and cytokinins were manually mapped and annotated using ARTEMIS (Berriman and Rutherford, 2003). Each ORF was screened based on an analysis of the GC frame plot of the reading-frames for each of the protein coding sequences (Bibb et al., 1984) and protein domains confirmed after comparison with those available in the Conserved Domains Database (CDD) of NCBI (Marchler-Bauer et al., 2015).

### Phylogenomic Analyses

The core genome of the *Frankia* strains was calculated using the default setting of BPGA 1.3 (Chaudhari et al., 2016) which identified 279 genes. The concatenated protein sequences of the core genes were aligned using MAFFT v7.300b (Katoh and Standley, 2013) and poorly aligned regions and missing data from the concatenated protein sequence alignments were removed using GBLOCKS (Castresana, 2000). The best-fit substitution model, LG+F+I+G4 was identified by ModelFinder (Kalyaanamoorthy et al., 2017) within the IQ-Tree algorithm (Nguyen et al., 2015), which was used to construct a maximum-likelihood dendrogram with 100,000 ultrafast bootstrap iterations and SH-like approximate likelihood ratio tests (Minh et al., 2013) from the resulting alignment.

## Results and Discussion

### Phylogenomic Diversity

**FIGURE 1:**
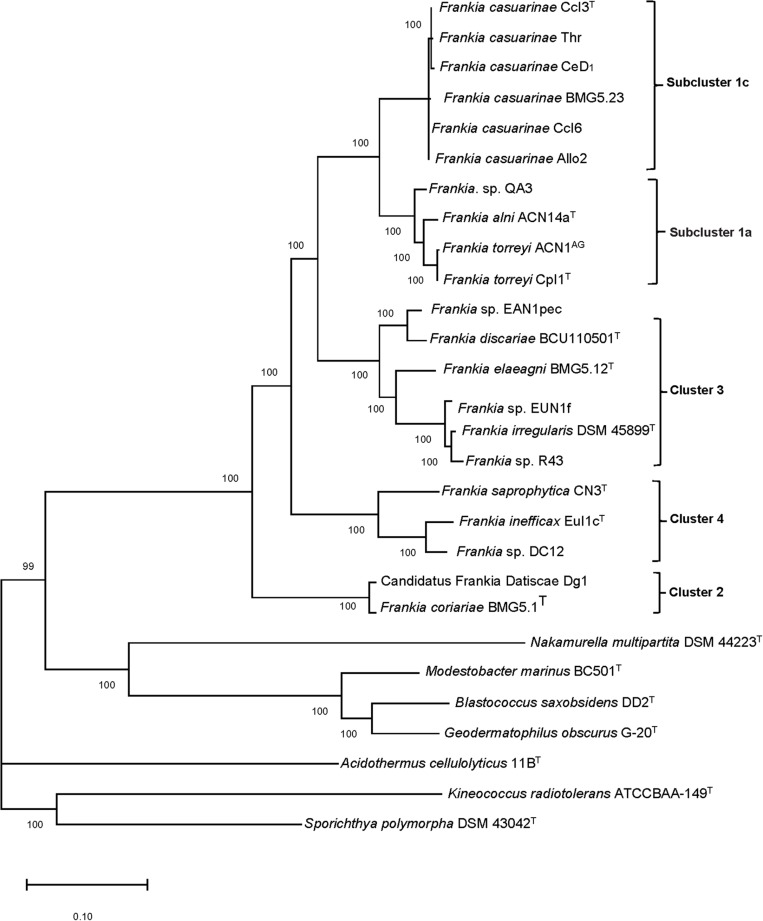
Maximum-likelihood dendrogram, based on 279 core proteins found among these genomes, with 100,000 ultrafast bootstrap iterations showing relationships between *Frankia* strains assigned to clusters.

The *Frankia* strains were assigned to four distinct clusters that were sharply separated from representatives of the seven related genera (Figure 1). Strains assigned to clusters 1 and 3 were found to have high genetic variability. Cluster 1 encompasses ten strains six of which were assigned to subcluster 1c, belonged to *F. casuarinae* (Gtari et al., 2019) while subcluster 1a was composed of four strains associated with *Alnus*–*Comptonia*–*Myrica*; the latter were assigned to three subgroups which enclosed *F. alni* ACN14a^T^, *Frankia* sp. QA3, and strains of *F. torreyi*. In turn, *Frankia* strain ACN1^AG^ has been classified as *F. torreyi* (Gtari et al., 2019). The topology of subclusters 1a and 1c is in line with that of the MLSA phylogenetic tree of Pozzi et al. (2018) where members of subcluster 1c, which show low genetic diversity, diverge from those of subcluster 1a while those a cluster 2 form a deep rooted evolutionary group. Cluster 3 strains were assigned to four subgroups containing (a) *Frankia* sp. EAN1pec and *F. discariae* BCU110501^T^; (b) *F. elaeagni* BMG5.12^T^; (c) *Frankia* sp. EUN1f; and (d) *F. irregularis* DSM 45899^T^ and *Frankia* sp. R43; all of the strains within this cluster form distinct species (Gtari et al., 2019). The overall group structures are highly supported and consistent with those represented by Pozzi et al. (2018). Minor differences in the topology of clusters 1 and 2 compared to the phylogenomic tree provided by Tisa et al. (2016), which was based on 1421 genes, are due to the diversity added by addition of more *Frankia* genomes and those of the related genera which reduced the core genome to 279 genes.

The *Frankia* strains classified in subclusters 1a and 1c showed genome sizes of 5–7.6 Mb and 5.0–5.4 Mb with gene numbers of 6287–6449 and 4350–5060, respectively. In turn, clusters 2 and 3 had genome sizes of 5.0–5.8 Mb and 7.5–10.4 Mb with 4799–5403 and 6845–8464 coding sequences, respectively. *Frankia* strains associated with cluster 4 had genome sizes within the range of 6.8–9.9 Mb with total gene numbers of 5743–8452 (Table 1). The genome sizes were found to be related to host specificity, found in previous studies (Normand et al., 2007; Tisa et al., 2016).

#### Direct Mechanisms

Free-living and symbiotic bacteria use direct and indirect mechanisms to promote and protect plant growth. The beneficial effect of *Frankia* strains in promoting plant growth has been the subject of several studies (Prat, 1989; Steele et al., 1989). Direct mechanisms include nitrogen fixation, phosphate solubilization, enhancement of mineral uptake, and phytohormone production.

##### Resource acquisition

###### Nitrogen fixation

Nitrogen is an essential element of most biomolecules that are crucial for life. It is available in the atmosphere as dinitrogen (N_2_) and can be converted into a plant-usable form through the activities of free-living diazotrophic microorganisms and mutualistic bacteria (Cleveland et al., 1999; Reed et al., 2011). These processes have important ecological and economical roles in sustainable agriculture.

The oxygen-labile enzyme, nitrogenase, converts atmospheric N_2_ into NH_3_. Microbes have developed different strategies to protect nitrogenase enzymes from oxygen inactivation. Free-living *Frankia* strains are able to fix atmospheric nitrogen independent of their host plant in specific cell structures named vesicles (Berry et al., 1993). *Frankia* vesicles, which contain nitrogenases, are surrounded by a lipid barrier that allows the enzyme to reduce dinitrogen (N_2_) to ammonium (${\mathrm{NH}}_{4}^{+}$) (Berry et al., 1993). *Frankia* strains in mutualistic associations with host plants are able to fix up to 300 N_2_ kg/hectare/year (Shantharam and Mattoo, 1997).

Nitrogenase complexes are composed of two major components: the first, the catalytic part of nitrogenase contains a Fe–Mo cofactor and P clusters (two iron-sulfur clusters) which are encoded by the structural genes *nif*D and *nif*K while component II is a nitrogenase reductase that comprises a Fe–S protein encoded by *nif*H (Dean et al., 1993; Hu et al., 2008). The *nif* operons consist of three structural genes (*nif*H, *nif*D, and *nif*K) and several accessorial genes such as *nif*V, *nif*E, *nif*N, *nif*X, *nif*W, *nif*Z, *nif*B, *nif*U, and *nif*S (Oh et al., 2003). Accessory genes have different roles in the maturation of inactive products, molecular scaffolds, and electron transport systems within nitrogenase complexes (Dos Santos et al., 2004). Three additional genes have been found within nitrogenase complexes: *or*A and *or*B genes encode for ferredoxin oxidoreductase alpha and beta units, respectively, while *fdx*I encodes for a ferredoxin (Souza et al., 2010). However, little is known about the distribution and organization of genes in the *nif* operons of *Frankia* strains (Oh et al., 2012).

**FIGURE 2:**
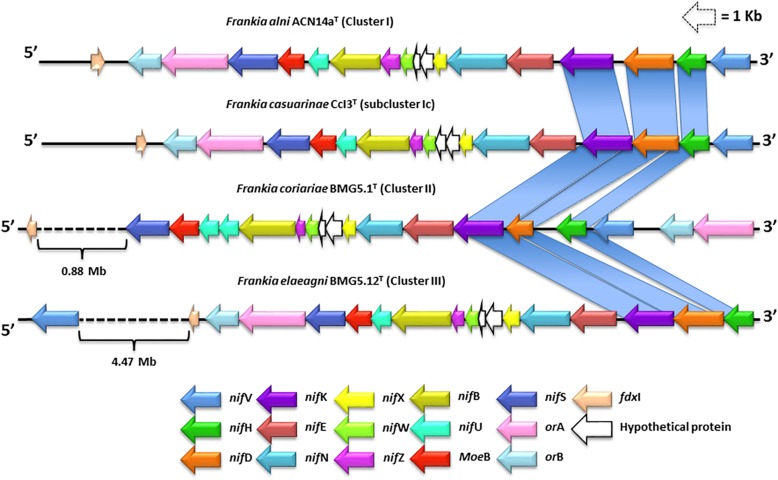
Genome mapping and comparative analysis of *nif* operons in strains representing *Frankia* subclusters 1a, 1c, 2, and 3. The *nif* operons are mainly composed of 18 well-conserved genes which enclose the structural *nif*HDK genes. The operon also contains the accessory genes, *nif*V, *nif*E, *nif*N, *nif*X, *nif*W, *nif*Z, *nif*B, *nif*U, and *nif*S.

In the present study, *nif* operons were found in the genomes of *Frankia* strains classified in clusters 1, 2, and 3 (Figure 1 and Supplementary Table S1). All of the accessory *nif* genes mentioned above, including *nif*HDK, were present in the genomes of *F. alni* ACN14a^T^, *F. casuarinae* CCI3^T^, *F. coriariae* BMG5.1^T^, and *F. elaeagni* BMG5.12^T^ (Figure 1). In addition, *nif*V genes were found in all of the *Frankia* genomes though in the case of *F. elaeagni* BMG5.12^T^ it was located 4.4 Mb downstream from the *nif* operon (Figure 2). In turn, *nif*V genes are considered to be essential for the activity of nitrogenase complexes because they encode for a homocitrate synthase that catalyzes the condensation of acetyl-CoA and α-ketoglutarate to homocitrate which is used as an organic component of the FeMo cofactor (Oh et al., 2003). However, the homocitrate synthase amino acid sequences of *Frankia* cluster 1 strains (26%), 2 (29%), and 3 (29%) are not closely related to those involved in the lysine biosynthesis pathways of yeasts and fungi. The alignment of homocitrate synthase amino acid sequences of *Frankia* strains with those of *Saccharomyces cerevisiae* showed low identity values between 26 and 29%. It is also interesting that *nif*ENX genes were clustered within the *nif* operon without any intergenic space (Figure 2). In addition, two orfs (1 and 2), which encode for the protein domains DUF269 and DUF68 with unknown function, were located between the *nif*X and *nif*W genes in all of the *Frankia* genomes (Figure 2). Finally, *or*A, *or*B, and *fdx*I genes were found in the genomes of all of the *Frankia* strains, as shown in Figure 2. However, the location of these genes was found to vary in the *nif* operon of *F. coriariae* BMG5.1^T^, here the *or*AB genes were located at the beginning of the operon upstream of *nif*V while *fdx*I was located approximately 0.9 Mb downstream of *nif*S (Figure 2).

###### Phosphate solubilization

Phosphorus (P) is an essential element in many biological processes including plant growth and, after nitrogen, is considered to be one of the most important elements limiting crop growth (Tak et al., 2012). Phosphate solubilizing microorganisms (PSM) are able to increase the bioavailability of P for plants by solubilizing inorganic phosphate (Zhu et al., 2011). To this end, microorganisms can release P from organic compounds either enzymatically (Rossolini et al., 1998) or by producing molecules, such as hydroxyl ions, CO_2_, organic acids, protons, and siderophores that solubilize inorganic phosphate (Rodriguez and Fraga, 1999; Sharma et al., 2013). The most effective PSM belong to the genera *Bacillus*, *Enterobacter*, *Flavobacterium*, *Micrococcus*, and *Rhizobium* and to the fungal taxa *Aspergillus* and *Penicillium* (Whitelaw, 2000). In contrast, little is known about the ability of representative *Frankia* strains to solubilize inorganic phosphate.

In the present study, the genomes of most of the *Frankia* strains were shown to contain an alkaline phosphatase gene (Supplementary Table S2) known to hydrolyze phosphomonoesters and catalyze the transfer of phosphoryl groups to alcohol in the presence of certain phosphate acceptors (Coleman, 1992). It seems likely that this gene is involved in mutualistic relationships between *Frankia* strains and their host plants by exchanging nutrients in a similar way to that suggested for arbuscular mycorrhizal associations (Aono et al., 2004). In addition, low-affinity inorganic phosphate transporter genes were found in the genomes of the *Frankia* strains belonging to cluster 1 and *F. asymbiotica* M16386^T^ (cluster 4). The alignment of amino acid sequences of the low-affinity inorganic phosphate transporter genes of *Frankia* showed identity values between 90.0 and 99.7% between *Frankia* strains of cluster 1 and 82.0% with strain M16386^T^. BLAST results of the alignment of amino acid sequences of low-affinity inorganic phosphate transporter genes of *Frankia* showed that they are closely related to those found in the genome sequences of other actinobacteria.

#### Phytohormones

Phytohormones have a crucial role in the growth, development, and the differentiation of plant tissues (Carro and Nouioui, 2017). The best-known ones are indole-3-acetic acid (IAA), cytokinins, ethylene (ET), and gibberellins; the levels of these hormones in plants can be regulated directly by soil microorganisms that synthesize these compounds.

##### IAA

It has been shown that PGP bacteria may have more than one biosynthetic pathway for the synthesis of hormones such as IAA (Mano and Nemoto, 2012). The latter can be synthesized *via* indole-3-acetamide (IAM) in phytopathogenic bacteria; the overproduction of IAA leads to the formation of plant tumors (Jameson, 2000). IAA can also be synthesized through the indole-3-pyruvic acid (IPA) pathway, directly by tryptophane (Trp) side chain oxidase (TSO) or through the indole-3-acetonitrile (IAN)/indole-3-acetaldoxime (IAOx) pathway (Glick, 2015). It is likely that L-tryptophan can be converted to IAM by tryptophan-2-monooxygenase that is encoded by the a*ux*1 gene, IAM is then transformed to IAA by IAM hydrolase following the expression of the *aux2* gene (Mano and Nemoto, 2012).

Several *Frankia* strains have been shown to produce auxins (Wheeler et al., 1984; Perrine-Walker et al., 2010) that are involved in *Frankia*–host plant interactions; decreased concentrations of auxins were found to have a negative effect on root nodule formation *in Casuarina glauca* (Hammad et al., 2003; Péret et al., 2007). The genomes of the type strains of *F. alni*, *F. casuarinae*, and *F. elaeagni* contain genes that have been seen to be involved in the IPA and phenyl pyruvate IAA biosynthetic pathways (Perrine-Walker et al., 2010) while the type strain of *F. discariae* has been found to produce IAA and gibberellins *in vitro* analyses (Solans et al., 2011).

In the present study, the genomes of all of the *Frankia* strains were shown to have genes that encode for indole-3-glycerol phosphate synthase which is considered to be a branch point of IAA in the tryptophan biosynthetic pathway in plants (Ouyang et al., 2000; Supplementary Table S3). This pathway requires the involvement of the gene products anthranilate phosphoribosyltransferase (*trp*D), anthranilate synthase, and aminase component (*trp*A and B) (Lambrecht and Downs, 2013) all of which were detected in the *Frankia* genomes.

##### Cytokinins

Cytokinins promote cell division and have growth regulatory functions in plants (Skoog and Armstrong, 1970). In general, they are formed by an adenine nucleotide together with an isoprene, modified isoprene, or aromatic side chain linked to a N^6^ amino group of adenine (Wong et al., 2015). These chemical structures are precursors to five types of cytokinins: trans-zeatin (tz), kinetin (K), N6-[2-isopentyl]adenine (iP), N6-benzyladenine (BA), and N6-isopentyladenosine (iPR) (Pertry et al., 2009).

The biosynthesis of cytokinins in plants and bacteria starts with the key intermediary dimethylallyl pyrophosphate (DMAPP), this isomerized form of isopentenyl pyrophosphate (IPP) is synthesized in the last step of the mevalonate pathway by isopentenyl-diphosphate delta isomerase (IDI) (Nett et al., 2017). In plants, an isopentenyl group from DMAPP is transferred to the N^6^ of ATP/ADP (Kakimoto, 2001) while bacteria start off with AMP, which is converted to an intermediary N6-isopentenyladenosine monophosphate (i6AMP) by isopentenyltransferase (ipt). i6AMP is the main enzyme responsible for the synthesis and expression of different variants of cytokinins (Kamínek et al., 1997); it is dephosphorylated to N6-iPR, the first active cytokinin, and is subsequently transformed to the second active cytokine, N6-iP, following an additional deribosylation step. In addition, i6AMP can be hydroxylated to generate the intermediary *trans*-zeatin riboside-5′-monophosphate (tZMP) which is subsequently dephosphorylated to produce *trans*-zeatin riboside (tZR) that undergoes deribosylation to yield the active cytokinin tz (Haberer and Kieber, 2002; Kakimoto, 2003; Sakakibara, 2006; Tarkowski et al., 2009; Frébort et al., 2011).

The *ipt* gene is common in the genomes of plant symbiotic bacteria, as exemplified by *Agrobacterium tumefaciens* where it is found in the T-region of the “Ti” plasmid which mediates infection in host plants while the homologous gene “*tzs*” is found near the *vir-*region on the same plasmid (Mok et al., 2000). Similarly, in *Rhodococcus fascians* D188^T^, a homologous gene *fas*D has been detected in the fas operon located on the pFiD188 plasmid which is involved in cytokinin biosynthesis and infection (Pertry et al., 2009, 2010).

**FIGURE 3:**
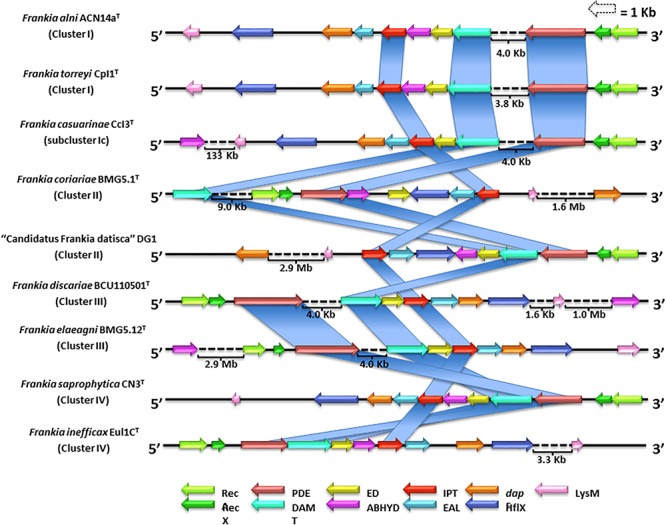
Genome mapping and comparative analysis of the putative cytokinin biosynthetic gene cluster for nine representative strains classified in the genus *Frankia*. Eleven well-conserved genes encode for: ispentenyltransferase (*ipt*); (dimethylallyl) adenosine tRNA methylthiotransferase (DAMT); phosphodiesterase (PDE); recombinase A (RecA) and its regulator (RecX); a lysin-motif (LysM); a pimeloyl-ACP methyl ester carboxylesterase (ABHYD); a cyclic diguanylate phosphodiesterase (EAL), a GTPase protein domain (HflX), an extradiol dioxygenase class III protein domain (ED), and a diaminopimelate epimerase (dapF).

Little is known about the ability of *Frankia* strains to produce cytokinins though *Frankia* strain HFPArI3 synthesizes iPR (Stevens and Berry, 1988). However, there is no clear evidence of the genetic mechanisms involved in the biosynthesis of cytokinins within *Frankia* strains. In the present study, genome mapping of cytokinin gene clusters in the nine strains that represented the *Frankia* clusters showed that they were composed of 11 highly conserved genes (Figure 3). Two of the genes were associated with the production of *ipt* and (dimethylallyl) adenosine tRNA methylthiotransferase (*damt*) (Figure 3) which are involved in the catalysis of the 2-methylthiolated derivative 2-methylthio-isopentenyladenosine (2MeSiPR) (Pertry et al., 2009). An additional gene in this putative cytokinin biosynthetic cluster encodes for a protein domain corresponding to a phosphodiesterase (PDE) that may be involved in the dephosphorylation of i6AMP to iPR. Most of the putative cytokinin biosynthetic gene clusters displayed two genes that encode for recombinase A (recA) and its regulator (RecX) which are involved in DNA exchange and homologous recombination (Roca and Cox, 1990; Kowalczykowski et al., 1994). A third gene located at the end of the gene clusters (Figure 3) encodes for a lysine-motif (LysM), a small protein domain found in bacteria and eukaryotes that is involved in signaling functions for plant–bacteria recognition during bacterial infections (Willmann and Nurnberger, 2012). These preliminary results not only provide a starting point for understanding cytokinin biosynthetic mechanisms in representatives of the genus *Frankia* but may also provide an insight into the process by which frankiae infect host plants.

Other genes observed in the putative cytokinin biosynthetic cluster encode for protein domains corresponding to genes that express for pimeloyl-ACP methyl ester carboxylesterase (ABHYD), a cyclic diguanylate phosphodiesterase (EAL); a GTPase protein domain (HflX); an extradiol dioxygenase (ED) class III protein domain; a PDE that may be involved in the dephosphorylation of i6AMP to form iPR (Levy et al., 2011); and a diaminopimelate epimerase (DapF) (Figure 3). At present, these are insufficient data to confirm the function of these genes in cytokinin biosynthesis. Clearly, further studies are required to determine the roles of these genes and the molecular mechanisms involved in cytokinin biosynthesis.

##### Ethylene

The positive effect of this gaseous hormone on plant growth (e.g., seed germination, formation of leaves, flowers, and fruits) is well known (Abeles et al., 1992; Bleecker and Kende, 2000). An increase in the production of ET in plants is a sign of biotic and abiotic stress (e.g., high salinity, increased temperature, insect predation, drought, flooding, presence of toxic compounds) which may lead to enhanced survival of plants or may trigger senescence when the stress persists and ethylene (ET) production is high (Glick, 2012). Methionine is the starting point for ET biosynthesis in plants via *S*-adenosyl-L-methionine (SAM) which is converted to 1-aminocyclopropane-1-carboxylic acid (ACC) in the presence of ACC synthase (ACS); ACC oxidase (ACCO) has a role in releasing ET and cyanide (converted to β-cyanoalanine to avoid toxicity in plants) (Yang and Hoffman, 1984). In diverse bacteria and fungi (e.g., *Escherichia coli*, *Cryptococcus albidus*), ET is synthesized through the oxidation of a transaminated derivative of methionine namely 2-*keto*-methylthiobutyric acid (KMBA) as well as by a lack of ammonia. In *Pseudomonas syringae* and *Penicillium digitatum*, the ET biosynthesis pathway calls for two substrates α-ketoglutarate and arginine which are calalyzed by an ET-forming enzyme (Eckert et al., 2014). In PGP rhizobacteria, ACC deaminase inhibits toxicity caused by high levels of ET in plants, it regulates ET levels by converting ACC produced by the plant to ammonia and α-ketobutyrate (Glick, 1995; GLick et al., 1998). Moreover, it has been shown that ACC deaminase has a significant role in the stimulation of the elongation of plant roots by PGP rhizobacteria. In this context, it is interesting that the genomes of all of the *Frankia* strains, apart from the *F. casuarinae* strains, contained genes associated with ACC deaminase (Supplementary Table S3).

#### Indirect Mechanisms

Plant growth promoting microorganisms also support the growth of plants by modulating environmental biotic and abiotic stress. They are able to either decrease, neutralize, or prevent infection of plants by phytopathogenic bacteria and fungi either by producing lytic enzymes or antibiotics (Singh and Jha, 2015; Gouda et al., 2018). These processes also support the growth of the plants under abiotic stress caused by drought, salinity, and extreme temperature (Akhgar et al., 2014).

##### Lytic enzymes

One of the defense strategies bacteria use against phytopathogenic fungi involves the production of hydrolytic enzymes such as cellulases, chitinases, glucanases, lipases, lysozymes, and proteases (Neeraja et al., 2010; Maksimov et al., 2011), as well as by other lytic compounds such as lactic acid. The most abundant insoluble polymer in nature, after cellulose, is chitin which can be hydrolyzed by chitinases ChiA, ChiB, and ChiC to *N*-*N*′-diacetylchitobiose which is converted to *N*-acetylglucosamine by *N*-acetylglucosaminidases. The genomes of *F. casuarinae* CcI3^T^, *F. inefficax* EuI1c^T^, *F. irregularis* DSM 45899**^T^**, and *F. saprophytica* CN3^T^ were found to contain genes which encode for chitinases whereas genes associated with cellulase production were only detected in the genomes of the type strains of *F. alni*, *F. torreyi*, and *Frankia* sp. ACN1^AG^ (Supplementary Table S4).

Three types of cellulases, endoglucanases (EC3.2.1.4), exoglucanases (EC3.2.1.91), and β-glucosidases (EC3.2.1.21), belonging to the glycosyl hydrolase family have been described. These enzymes, which are present in microorganisms isolated from diverse ecological niches (Lynd et al., 2002), transform cellulose to glucose. They are also active against phytopathogenic fungi since they hydrolyze β-1,3-glucan, the principal component of fungal cell walls, and indirectly stimulate plant defenses by releasing immune elicitors from the cell walls (Lynd et al., 2002).

The genomes for all of the *Frankia* strains, apart from those of the type strains of *F. casuarinae*, *F. inefficax* and *F. irregularis*, were shown to contain a gene encoding for an endoglucanase that has an important role in initiating cellulose hydrolysis (Supplementary Table S4; Cohen et al., 2005). In addition, the cluster 3 strains and the type strain of *F. saprophytica* (cluster 4) were found to have the capacity to produce an extracellular endoglucanase which has been detected in *Paenibacillus polymyxa* BEb-40 (Gastelum-Arellanez et al., 2014) and used in industry to breakdown lignocellulose (Supplementary Table S4). Furthermore, a gene encoding for the type III effector *hrp*W hairpin, known to induce hypersensitivity responses in plants (Charkowski et al., 1998) and previously detected in plant-related actinobacteria (Carro et al., 2018), was detected in the genomes of *F. elaeagni* BMG5.12^T^ and *F. saprophytica* CN3^T^ (Supplementary Table S4). Gene *pl*, which encodes for pectate lyase (PL), was detected in the genomes of *F. saprophytica* CN3^T^ and *Frankia* strains assigned to cluster 3. This gene has been found in pathogenic bacteria and is known to degrade host tissues, a process in line with its role in the maceration and soft rotting of plant tissues (Marín-Rodríguez et al., 2002). Since the gene *hrp*W is associated with PA production, it seems likely that genes *hrp*W and *pa* are involved in the initiation of *Frankia*–host plant interactions.

##### Siderophores

Iron is an essential element for all organisms, including microorganisms. Bacteria and fungi produce siderophores in response to iron limitation (Saha et al., 2016). Consequently, these Fe^3+^ chelators have an important role in the survival of bacteria, including pathogens, by scavenging iron from iron-binding proteins produced by their hosts (Wandersman and Delepelaire, 2004). The genomes of the *F. casuarina* strains were shown to harbor a gene that encodes for 2-amino-3,7-dideoxy-D-threo-hept-6-ulosonate synthase (*aro*A′) which is involved in the shikimate pathway (Supplementary Table S3). Chorismate synthase (CS), chorismate mutase (CM), and shikimate synthase are known to be fundamental in catalyzing the aromatic amino acid (AAA) biosynthetic pathway which is necessary for the production of specialized metabolites essential for plant growth (Helmstaedt et al., 2001; Sasso et al., 2004). The AAA, chorismate, is considered to be an intermediate compound from which catecholate siderophore is synthesized, a reaction that involves a series of enzymes (Walsh et al., 1990). The expression of siderophore genes is regulated by an iron-binding repressor protein, a ferric uptake regulator (Fur) (Escolar et al., 1999), which is common in Gram-negative and AT-rich Gram-positive bacteria; the genome of the GC-rich actinobacterium, *Corynebacterium diphtheriae*, contains a diphtheria toxin repressor (*dtxR*) which is essential for siderophore-dependant iron uptake (Qian et al., 2002). Several siderophores have been described in actinobacteria, such as desferrioxamine (G, B, and E), tsukubachelin, and oxachelin, which are characteristic of *Streptomyces* species (Challis and Hopwood, 2003). In addition, catecholic and hydroxamate moieties have been detected in 44% of soil actinobacteria (Nakouti et al., 2012) while heterobactin has only been reported from *Nocardia* and *Rhodococcus* strains (Lee et al., 2012; Wang et al., 2014).

The genomes of all of the *Frankia* strains showed some variation in the distribution of genes involved in the production of siderophores though siderophore biosynthesis non-ribosomal peptide synthetase modules were found in all of the *Frankia* genomes; siderophore biosynthesis proteins, related to a monooxygenase and to diaminobutyrate–2-oxoglutarate amino transferase, were present in all of the *Frankia* genomes except those of the cluster 4 strains (Supplementary Table S5).

##### Stress genes

Bacteria have developed several ways of coping with environmental stress. In this context, they produce three types of hemoglobin proteins: truncated hemoglobins (trHbo), hemoglobins (Hbos), and flavohemoglobins (flavoHbo), in response to oxygen limitation, oxidative and nitrosative stress. *Frankia* strains produce two of these hemoglobins: Hbo and flavoHbo. There are two types of trHbo, namely HboO and HboN, which act as scavengers of O_2_ and NO, respectively (Frey and Kallio, 2003; Supplementary Table S6) while flavoHbo is involved in the nitric dioxygenase reaction by detoxifying NO and protects bacteria from several noxious nitrogen compounds (Frey and Kallio, 2003). The genomes of several *Frankia* strains express for trHBo- and flavoHbo-associated products that may protect them from nitrosative stress and increase their respiration rates in low-oxygen environments (Beckwith et al., 2002; Tjepkema et al., 2002; Niemann et al., 2005; Niemann and Tisa, 2008). These genes are expressed when host plants are infected followed by the liberation of free radical oxygen and nitric oxide which act as plant defense mechanisms (Niemann and Tisa, 2008).

In this present investigation, the genomes of *Frankia* strains classified in subcluster 1a and some representatives of subcluster 1c (strains CcI3, CeD, and BMG5.23) and cluster 3 (strains EUN1f and R43) were shown to carry the *hmp*X gene which encodes for a flavohemoglobin involved in nitrosative stress (Supplementary Table S6). In addition, *hbo*N and *hbo*O genes were detected in the genomes of *F. alni* ACN14a^T^, *F. torreyi* CpI1^T^ (subcluster 1a), *F. casuarinae* CcI3^T^ (subcluster 1c), *F. discariae* BCU110501^T^, *F. elaeagni* BMG5.12^T^ (cluster 3), and *F. inefficax* Eul1C^T^ (cluster 4). Interestingly, only the genomes of *F. coriariae* BMG5.1^T^, candidatus *Frankia datiscae* Dg1 (cluster 2), and *F. saprophytica* CN3^T^ (cluster 4) contained the *hbo*O gene which is involved in hypoxic stress. All of these results are in good agreement with those from previous studies (Beckwith et al., 2002; Niemann et al., 2005; Niemann and Tisa, 2008).

The presence of such putative stress genes in *Frankia* strains was expected since they are known for their ability to survive in harsh environments, including nutrient poor soils (Karthikeyan et al., 2009; Oshone et al., 2017). Further, the genomes of all of the *Frankia* strains carried a common set of genes, notably ones associated with the production of alkyl hydroperoxide reductase (*ahp*) which is involved in hydrogen peroxide stress (Bsat et al., 1996) and in the defense of DNA against oxidative damage (Jacobson et al., 1989); the peroxide stress regulator *per*R, which is related to the FUR family; redox-sensitive transcriptional regulators (*rex* and *sox*) (Wietzke and Bahl, 2012) that have a role in oxidative stress protection; rubrerythrin (*rbr*), which encodes for a peroxidase and has a role in the protection of nitrogenase from oxygen in cyanobacteria (Zhao et al., 2007); aquaporin Z (*aqp*Z), which is associated with drought stress and cold (*csp*A and C) and heat shock (*grp*E) and chaperon proteins (*dna*J and K) that are involved in heat shock responses (Paek and Walker, 1987; Ellis and Hemmingsen, 1989) and the zinc uptake regulator protein (*zur*) which helps to protect bacteria against oxidative stress (Smith et al., 2009).

Genes encoding for L-proline glycine betaine binding ABC transporter proteins (*pro*X and V) play a crucial role in resistance to osmotic stress in Gram-negative bacteria, such as *Sinorhizobium meliloti* (Le Rudulier and Bernard, 1986) were found in all of the *Frankia* genomes, apart from those of subcluster1c and cluster 2 strains (Supplementary Table S6). This finding is consistent with the observation of Oshone et al. (2017) who noted the absence of sarcosine oxidase (SO) genes in *F. casuarinae* strains.

All of the *Frankia* genomes were found to contain a range of genes associated with DNA repair systems, as exemplified by exconuclease *ABC* (*uvr operon*) and formamidopyrimidine-DNA glycosylase (Gly1) which are responsible for the oxidation of purines of damaged DNA (Supplementary Table S6). Similarly, all of the genomes harbored genes that encode for enzymes involved in photosynthesis, such as phytoene synthase (*crt*B) and octaprenyl diphosphate synthase (*isp*B) (Supplementary Table S6). Genes associated with carotenoid biosynthesis (e.g., β-carotene ketolase) were detected in the genomes of some of the *Frankia* strains belonging to clusters 1 and 4 (Supplementary Table S6). Carotenoids have a crucial role in preventing photooxidative damage (Howitt and Pogson, 2006) and are considered to be precursors of abscisic acid, a phytohormone involved in the control of water retention and some other stress responses (Koornneef, 1986). Further, the genomes of the *F. casuarinae* strains and those of the representatives of cluster 4 contained the NAD(P) transhydrogenase gene (Supplementary Table S6), which is involved in the reduction of glutathione, an antioxidant that has an important role in preventing damage to cellular components caused by reactive oxygen species (Pompella et al., 2003).

In addition to the ability of *Frankia* strains to solubilize and convert insoluble phosphate to bioavailable forms, some of them are able to modulate the lack of phosphate in natural environments. In this context, several genes that encode for inducible phosphate starvation (*psi*), and which belong to the PHO regulon (Hsieh and Wanner, 2010), are involved in organic phosphate solubilization and uptake by either enhancing the ability of cells to efficiently use limited sources of phosphate or to provide access to other sources of phosphate (Antelmann et al., 2000). The genomes of all of the *Frankia* strains were found to contain *phoA*, *phoB*, *pho*H, *pho*R, *pho*U, *phy* (phytase), *tag*, *ush*A (nucleotidase), and *pts*ABCS genes (Supplementary Table S2). The *pho*A and *pho*B genes encode for alkaline phosphatase while *pho*D expresses for PDE/alkaline phosphatase D which has a role in teichoic acid turnover in the cell wall in *Bacillus subtilis* (Eder et al., 1996); the *pst*S gene belongs to the *pst*SACB1B2 operon which is involved in phosphate transport (Eymann et al., 1996; Qi et al., 1997).

The alkaline phosphatase genes identified in *Frankia* strains have amino acid sequence similarities of 53–58% and are similar to those found in some actinobacterial species. However, the alignment and comparison of alkaline phosphatase of *Frankia* strains to PhoA, PhoC, and PhoD proteins of *Streptomyces coelicolor* showed identity values between 41.9–47, 41.9–54.8, and 26.5–28.5%, respectively, and 35.0–46.4% with the *pho*A gene from *Streptomyces griseus*. The alkaline phosphatase of *F. elaeagni* BMG5.12^T^ showed an amino acid sequence identify value of 58.3% with the *pho*C gene. These results show that the alkaline phosphatases of *Frankia* strains are quite specific and are not closely related to the well-studied ones of the cited *Streptomyces* species.

### Overview, Significance, and Future Studies

*Frankia* strains are well known for their ability to form nitrogen-fixing nodules in actinorhizal plants and to promote plant growth. Genome mining of representative *Frankia* strains representing the four host infection groups not only show that the genetic machinery of their nitrogenase complexes are conserved but also highlighted the presence of 11 conserved genes (*ipt*, *damt*, *rec*A, *rec*X, *lysM*, *eal*, *hfl*X, *ed*, *dap*F, *pde*, and *abhyd*) in the putative cytokinin biosynthetic gene cluster; the presence of the LysM domain and recombinase genes indicates that the cytokinin cluster may also be involved in the ability of *Frankia* strains to infect their hosts plant. In addition, the genomes of all of the *Frankia* strains were shown to be equipped with genes associated with the synthesis and production of phytohormones and contained genes functionally linked to inorganic phosphate solubilization and siderophore production. Moreover, the genomes of all the representative strains carried a set of universal genes the products of which are involved in modulating the effects of abiotic and biotic environmental stress. Consequently, it can be concluded that *Frankia* strains should be seen as potential substitutes for chemical fertilizers and thereby may prove to have an important role in the improving ecosystem quality. However, further work is required to understand the PGP mechanisms of frankiae before they can be developed for use in sustainable agriculture.

## Data Availability

The datasets analyzed for this study can be found in the National Center for Biotechnology Information: https://www.ncbi.nlm.nih.gov/.

## Author Contributions

IN conceived the project and performed the genome mining analyses while IN and MiG developed the concepts. VS carried out the phylogenomic analyses and interpreted the results together with IN, MiG, LT, and H-PK. CC-A, LC, JFC, H-PK, FG-G, MaG, LT, and VS played roles in analyzing the data and in interpreting the results. IN and MiG wrote the manuscript. All the authors approved the final version.

## Conflict of Interest Statement

The authors declare that the research was conducted in the absence of any commercial or financial relationships that could be construed as a potential conflict of interest.
